# COVID-19 Antibody Detecting Rapid Diagnostic Tests Show High Cross-Reactivity When Challenged with Pre-Pandemic Malaria, Schistosomiasis and Dengue Samples

**DOI:** 10.3390/diagnostics11071163

**Published:** 2021-06-25

**Authors:** Fien Vanroye, Dorien Van den Bossche, Isabel Brosius, Bieke Tack, Marjan Van Esbroeck, Jan Jacobs

**Affiliations:** 1 Department of Clinical Sciences, Institute of Tropical Medicine, 2000 Antwerp, Belgium; dvandenbossche@itg.be (D.V.d.B.); ibrosius@itg.be (I.B.); btack@itg.be (B.T.); mvesbroeck@itg.be (M.V.E.); jjacobs@itg.be (J.J.); 2 Department of Microbiology, Immunology and Transplantation, KU Leuven, 3000 Leuven, Belgium

**Keywords:** SARS-CoV-2, COVID-19, cross-reactivity

## Abstract

COVID-19 Antibody Detecting Rapid Diagnostic Tests (COVID-19 Ab RDTs) are the preferred tool for SARS-CoV-2 seroprevalence studies, particularly in low- and middle-income countries. The present study challenged COVID-19 Ab RDTs with pre-pandemic samples of patients exposed to tropical pathogens. A retrospective study was performed on archived serum (*n* = 94) and EDTA whole blood (*n* = 126) samples obtained during 2010–2018 from 196 travelers with malaria (*n* = 170), schistosomiasis (*n* = 25) and dengue (*n* = 25). COVID-19 Ab RDTs were selected based on regulatory approval status, independent evaluation results and detecting antigens. Among 13 COVID-19 Ab RDT products, overall cross-reactivity was 18.5%; cross-reactivity for malaria, schistosomiasis and dengue was 20.3%, 18.1% and 7.5%, respectively. Cross-reactivity for current and recent malaria, malaria antibodies, *Plasmodium* species and parasite densities was similar. Cross-reactivity among the different RDT products ranged from 2.7% to 48.9% (median value 14.5%). IgM represented 67.9% of cross-reactive test lines. Cross-reactivity was not associated with detecting antigens, patient categories or disease (sub)groups, except for schistosomiasis (two products with ≥60% cross-reactivity). The high cross-reactivity for malaria, schistosomiasis and—to a lesser extent—dengue calls for risk mitigation when using COVID-19 Ab RDTs in co-endemic regions.

## 1. Introduction

COVID-19 antibodies confirm current and past infection by SARS-CoV-2 infection. COVID-19 Ab diagnostics have limited relevance for individual patient care [1,2,3,4], but are valuable at the public health level for seroprevalence studies. Seroprevalence studies provide information about the extent of the epidemic, case fatality rates and the risk groups affected [1,2,3,4,5,6].

A plethora of COVID-19 Ab Detecting Rapid Diagnostic Tests (COVID-19 Ab RDTs) are marketed—at the time of writing (May, 2021), the diagnostic tracker of the Foundation of Innovative Diagnostics (FIND) lists 213 products [7]; most are lateral-flow immunoassays (LFA) based on a nitrocellulose reagent strip housed in a plastic cassette. Compared to laboratory platforms (enzyme immunoassays (EIA) and chemiluminescence assays (CLIA)), COVID-19 Ab RDTs have simple logistics, require relatively little training, have a short turnaround time, are easily scalable and are amenable to self-testing. As such, they are therefore preferred tools for seroprevalence studies [5,8,9], despite the fact that the WHO recommends the use of enzyme immunoassays for seroprevalence studies [1]. In low- and middle-income countries (LMICs), COVID-19 Ab RDTs have also been deployed for triage, diagnosis and contact tracing [10,11,12].

Specificity testing of COVID-19 Ab RDTs so far has focused on seasonal coronaviruses (NL63, HKU1, 229E, OC43), SARS-CoV, cytomegalovirus, Epstein–Barr virus, human hepatitis B virus, *Mycoplasma pneumoniae* and parvovirus infection in addition to interfering conditions (rheumatoid factor, autoimmune pathologies and hyperglobulinemia) [8,13,14,15,16,17,18,19,20,21,22,23,24,25,26]. Tropical diseases such as malaria, dengue, schistosomiasis and human African trypanosomiasis are well-known for their cross-reactivity with HIV-1 antibody LFA RDTs and malaria antigen RDTs [27,28,29,30]. The World Health Organization (WHO) has listed malaria and dengue among the organisms to be tested for cross-reactivity of COVID-19 serological tests [31,32]. The present study assesses COVID-19 Ab RDTs for cross-reactions when challenged with pre-pandemic samples of patients with malaria, schistosomiasis and dengue.

## 2. Materials and Methods

This retrospective reference study was conducted by the WHO Collaborating Centre for HIV/AIDS Diagnostics and Laboratory Support and (re) emerging viral infections and malaria (BEL-27) at the Institute of Tropical Medicine (ITM), Belgium. COVID-19 Ab RDTs were selected based on design (LFA platform, visual reading), regulatory approval, independent product evaluations [33,34] and representation of different detecting antigens. A comparator enzyme immune assay detecting IgG and IgM antibodies was selected based on the low required sample volume (10 µL) and separate detection of IgG and IgM. At the stage of selection and procurement, the only EIA product available that met both criteria was the DiaPro IgG and IgM EIA (Launch Diagnostics Ltd., Sesto San Giovanni, Italy). Table 1 lists the products, manufacturers, detecting antigens used, claimed specimen type and the shortened product names used in the text. Supplementary Table S1 provides full information about the products. Most manufacturers had only one lot available, and testing was limited to a single lot per product.

In total, 220 archived left-over samples (stored at −80 °C) were selected from 196 patients with malaria, schistosomiasis and dengue who had consulted the travel clinic of ITM between 2010 and 2018. Table 2 lists the diseases and their diagnostic selection criteria [27,35,36,37], specimens (EDTA anticoagulated whole blood and serum), as well as demographic data and travel destination or country of the patients. Supplementary Table S2 provides demographic and geographic details of the samples. Malaria samples comprised the four main *Plasmodium* species, a range of different parasite densities of *P. falciparum* and disease subgroups (current and recent malaria, presence of malaria antibodies). Ethical clearance was obtained from the Institutional Review Board of ITM (06-2020) and the samples were registered and stored in the ITM Biobank, according to the Belgian Biobank legislation (06-2020).

Before study initiation, samples were aliquoted in smaller volumes to limit freeze–thaw cycles. Pilot testing preceding formal assessment included testing of a serum and ETA whole blood sample from a SARS-CoV-2 Ab negative and positive control patient, and a commercial COVID-19 negative and positive control sample (Multichem ID-COVID19 Neg control and ID-COVID19G/M control, Zentech, Angleur, Belgium) (Supplementary Table S3).

Biomedical staff experienced in RDT evaluations performed testing in batches of 10 tests. Operators were blinded to the sample’s identification and followed the manufacturer’s procedure as described in the instructions for use, except for the use of a micropipette instead of the product’s sample device. Two operators independently read the test results at the end of the prescribed RDT readout time, alternating first and second reader. Invalid results were defined as absence of control line or no visibility of control line caused by errors in migration and background clearance; these anomalies were grouped as previously described for malaria RDT testing [38]. A standardized grading scale for line intensity was used [39]: line intensities included very faint, faint, weak, medium and strong. Ghost lines (colorless shade of lines caused by impression of the nitrocellulose strip at the application of the test and control line antibodies) were disregarded. In case of discrepant categorical results (positive, negative or invalid), reading by a third observer was used to obtain a final result. Immediately after reading, photographs were taken.

Results for the DiaPro IgG and IgM EIA were expressed as a ratio of the sample optical density (OD) 450 nm/620–630 nm and the index values (S/CO, S = ratio of the sample, CO = mean value of three negative controls plus a value of 0.250). Interpretations were negative if S/CO was <0.9, grey-zone if S/CO was between 0.9 and 1.1, positive if S/CO >1.1. Grey-zone results were considered as positive results. Comparison between EIA IgM index values and RDT IgM cross-reactivity was calculated with Wilcoxon rank sum test in R (https://www.r-project.org/, Vienna, Austria).

Data were recorded in an Excel database (Microsoft Office Windows 10, Richmond, Virginia, USA). A visible line of any category of line intensity was considered as positive (cross-reactive). When considering the denominator for calculation, invalid test results were subtracted. Overall cross-reactivity was expressed as the proportion of cross-reactive samples with any test line positive (IgG, IgM or total immunoglobulin (total Ig)) among all tests performed. Cross-reactivity was further calculated for subgroups of samples, specimen types, immunoglobulin classes and patient categories. For subgroups of *Plasmodium* species and *P. falciparum* parasite densities, cross-reactivity for at least one RDT product was used for comparison. For comparison of line intensities, results read by the first observer were used [38] and the denominator was the total of cross-reactive test lines (IgG, IgM, total Ig). For proportions of cross-reactivity, 95% confidence intervals (95% C.I.) were calculated and statistical significance was assessed by the chi-square test or in case of low samples the Fisher Exact test. Categorical agreement between observers was calculated with Cohen’s kappa with package “fmsb” in R (https://www.r-project.org/, Vienna, Austria, accessed on 18 May 2021), and invalid results were excluded.

## 3. Results

A total of 13 COVID-19 Ab RDT products were assessed. Twelve RDT products had separate IgG and IgM test lines with the IgM test line being the most proximal; the remaining (Wantai) did not specify a particular class of immunoglobulins, and it was categorized as “total immunoglobulins” (total Ig). StrongStep had separate reagent strips for IgM and IgG but embedded in one cassette. All 13 products mentioned serum, plasma and whole blood as claimed specimens, and seven products mentioned not to use frozen blood. Five products mentioned to consider any shade of color as a positive line.

Detecting antigens were mentioned in the manufacturer’s instructions for use for only two RDT products (QuickZen, Angleur Belgium and Dynamiker, Tianjin, China); the remaining information was retrieved from regulatory documents. They comprised spike (*n* = 2) and nucleocapsid proteins (*n* = 4), the receptor binding domain (*n* = 1) and combinations of antigens (*n* = 6); the DiaPro EIA used spike protein for IgG and both spike and nucleocapsid protein for IgM. According to the instructions for use, RDT products’ specificity for IgG/IgM combined ranged from 92.8% (PanBio) to 100% (Toda).

A total of 220 samples were tested, representing malaria (77.3%), schistosomiasis (11.4%) and dengue (11.4%) (Table 2). Specimens were serum (*n* = 94, 42.7%) and EDTA whole blood (*n* = 126, 57.3%), the latter mostly (94.4%) from malaria patients. Samples had been obtained from 196 patients, and for 24 patients both EDTA and serum samples were available. Median age (interquartile range) was 35 (24–47) years, 27 (13.8%) were children (≤15 years old) and 63.3% were male. Most (*n* = 23, (11.7%)) were outpatients. From the 23 (11.7%) patients who had been hospitalized, most (20/23; 87.0%) were suffering from malaria. Geographic origin or travel destination (available for 179 patients (91.3%)) was mostly Africa (86.0%), except for patients with dengue. Categories of patients (available for 117 (59.7%) patients) comprised expatriates and travelers (*n* = 51, 43.6%) and visiting friends and relatives and migrants (*n* = 66, 56.4%).

All RDT products generated visible test lines with the patient positive control, but three products had very faint test lines. The Multichem ID-COVID19G/M control revealed positive IgG lines for all but one products; for the IgM lines, four products had no visible test lines, whereas five out of nine visible test lines were very faint (Supplementary Table S3). Seven out of ten buffer vials of Cellex showed a green discoloration; culture revealed abundant growth of *Pseudomonas aeruginosa*. By consequence, Cellex could only be assessed for 158/220 (71.8%) samples. Invalid test results were observed for 71/1625 (4.4%) and 1/1390 (0.07%) EDTA blood and serum sample/test combinations, respectively (*p* < 0.001). Seven RDT products had invalid test rates below 1%, and five products had rates between 1 and 5%. The product mentioning not to use frozen blood (QuikZen) had an invalid test result rate of 19.1% (Supplementary Table S3).

Overall (for all RDT products and samples combined), cross-reactivity was observed in 18.5% (517/2794) of tests performed (for details including 95% C.I., see Table 3). Among the RDT products, cross-reactivity varied widely; the median (range) of overall cross-reactivity among RDT products was 14.5% ranging from 2.7% (Toda) to 48.9% (Boson). Cross-reactivity for serum samples (20.8%, 250/1202) was higher compared to EDTA blood samples (16.8%, 267/1592; *p* = 0.007). When both EDTA and serum samples were available (*n* = 24 pairs), cross-reacting IgM lines were more frequent in serum samples (17.9%, 56/312) compared to EDTA blood samples (6.4%, 20/312; *p* < 0.001); for the IgG line, the tendency was the same (2.9% (9/312) versus 1.0% (3/312); *p* = 0.008).

For all 13 RDT products combined, cross-reactivity was 20.3% for malaria and 18.1% for schistosomiasis, but lower for dengue (7.5%; *p* < 0.001). Singuway and Healgen had high cross-reactivity for schistosomiasis (60.0% and 76.0%, respectively), versus a maximum of 20% for the other RDT products. The highest proportions of cross-reactivity for malaria and dengue were observed for Boson (56.2% and 32.0%, respectively). There was no apparent association between cross-reactivity and detecting antigens used in the RDT products. Overall cross-reactivity was similar for both patient categories, i.e., 10.0% for expatriates and travelers (*n* = 1464) versus 10.7% for visiting friends and relatives and migrants (*n* = 1902). This was also observed for IgG and IgM test lines separately: 3.7% and 15.2% for the category expatriates and travelers (*n* = 702) versus 4.0% and 16.3% for the category visiting friends and relatives and migrants (*n* = 912).

Among malaria sub-groups (current malaria, recent malaria, presence of malaria antibodies), cross-reactivity was similar: 19.2%, 24.4% and 21.6% (*p* = 0.119) (Table 3). Cross-reactivity was also similar for the different *Plasmodium* species: most samples with *P. falciparum* (86/95, 90.5%), *P. vivax* (11/11), *P. ovale* (9/12) and *P. malariae* (7/8) showed cross-reactivity with at least one RDT product. Likewise, cross-reactivity for at least one RDT product was similar for the *P. falciparum* parasite density groups (Table 4).

On 545 cross-reactive sample–product combinations, 67.9%, 8.1% and 15.6% showed either IgM, IgG or total Ig test lines, respectively (8.4% showed both IgM and IgG lines) (Supplementary Table S4). All RDT products had higher cross-reactivity for IgM compared to IgG but proportions varied; in four products, IgG represented <10% of cross-reactive lines. Among cross-reacting IgM lines (*n* = 370), 70.0% were very faint or faint and 23.5%, 6.2% and 0.3% had weak, medium and strong line intensities, respectively. For IgG, these proportions were 60.0% (very faint/faint), 30.0% (weak), 7.8% (medium) and 2.2% (strong). The Wantai had exclusively faint or very faint line intensities.

Categorical agreement (negative, positive and invalids) between observers 1 and 2 for IgM and IgG lines was, respectively, 93.5% (2612/2793) and 96.2% (2686/2793). For all cross-reactive readings combined, agreement within one category of line intensity was 69.9% (144/206) for IgG and 71.8% (382/532) for IgM. An almost perfect to perfect agreement for IgG and IgM (kappa value 0.82–1.0) was found for Healgen, Toda, Boson, StrongStep, Cellex and Multi-G.

The DiaPro EIA cross-reactivity for IgG occurred in 1.8% (*n* = 2) of samples, and for IgM it occurred in 16.2% (*n* = 18) of samples, respectively. Both IgG positive samples occurred with malaria samples and were clearly positive (S/CO values of 1.73 and 8.69, respectively). For IgM (*n* = 18), cross-reactions occurred with malaria (*n* = 13), dengue (*n* = 3) and schistosomiasis (*n* = 2); ten and eight were positive and grey-zone positive, respectively. There was an association between EIA IgM index values and RDT IgM cross-reactivity for Toda, Boson, Biohit, Abbott, Dynamiker, Cellex and Multi-G (*p* ≤ 0.01).

For some products, issues of usability were noted, including peeling labels (160/220 outer packages (QuickZen)) and incorrectly assembled cassettes (shifted test strips, incompletely closed cassettes: QuickZen (*n* = 17), Cellex (*n* = 15) and StrongStep (*n* = 4)). In addition, weak intensity control lines were observed for Healgen (3.2%), StrongStep (4.1%), QuickZen (27.3%) and SureScreen (45.0%).

## 4. Discussion

The present study showed, for 13 COVID-19 Ab detecting RDTs, an overall cross-reactivity of 18.5% when tested with pre-pandemic samples of patients with malaria, schistosomiasis and dengue; overall cross-reactivity for these diseases was 20.3%, 18.1% and 7.5%, respectively. Cross-reactivity in malaria subgroups (current and recent malaria, malaria antibodies), species and parasite densities was similar. Apart from the high (≥60%) cross-reactivity of two products when tested with samples from patients with schistosomiasis, no particular associations of cross-reactivity were observed for (sub)group of diseases, detecting antigen used and patient’s categories. Among the RDT products, cross-reactivity varied widely; the median (range) of overall cross-reactivity was 14.5% (2.7%–48.9%). IgM represented two-thirds (67.9%) of overall cross-reactive test lines versus 16.6% and 15.6% for IgG and total Ig lines. A total of 30% and 40% of IgM and IgG test lines had at least a weak line intensity, i.e., were clearly and unequivocally visible. The comparator EIA showed lower proportions of cross-reactivity (1.8% and 16.2% of IgG and IgM test lines).

Clinical observations [40] and seroprevalence studies have put forward malaria as a potential cause of cross-reactivity among COVID-19 Ab RDTs, potentially explaining the unexpected high COVID-19 test reactivity in sub-Saharan Africa [41]. Diagnostic studies on pre-pandemic samples provided evidence in favor of cross-reactivity by *P. falciparum* in COVID-19 Ab EIA and RDTs [18,19,42,43], but sample sizes were small, associations did not reach statistical significance and findings were not consistent [14]. The present study confirmed that current malaria, recent malaria and the presence of malaria antibodies can cause cross-reactivity in COVID-19 Ab RDTs. Cross-reactivity occurred across *P. falciparum* parasite density groups and also among the non-falciparum *Plasmodium* species.

Likewise, previously published clinical observations pointed to the cross-reactivity of dengue infection [44]. Two studies (one from Indonesia, another from Italy) revealed low rates of cross-reactivity (≤1/60 and 1/44 samples) for five and two RDT products tested with dengue positive serum samples [25,45]. Of note, for two products assessed in the Indonesia study (Cellex and Dynamiker), the present study showed 16% and 5% cross-reactivity, respectively. Variable results were obtained for COVID-19 Ab EIA platforms [16,46]: a study from Colombia did not observe any cross-reactivity among 23 pre-pandemic serum samples from patients with acute or recent dengue infection [16], whereas for another EIA platform, 22% cross-reactivity was observed among 95 pre-pandemic serum samples of dengue-infected travelers with the Euroimmun IgA/IgG EIA assay [46].

Schistosomiasis is known as a cause of cross-reactivity in HIV antibody RDTs and *P. falciparum* antigen detecting RDTs [28,47], but to our knowledge schistosomiasis has not been assessed for cross-reactivity with COVID-19 Ab. The present study showed an overall 18% cross-reactivity rate for *Schistosoma* antibody positive samples, reaching ≥60% for two RDT products (Singuway and Dynamiker).

The large variation in cross-reactivity among RDT products in the present study and the absence of clear association with disease (sub)groups and detecting antigens point to a non-specific mechanism of cross-reactivity. *Plasmodium*, *Schistosoma* and dengue virus are known to trigger polyclonal B-cell activation with the production of antibodies that can cause false-positive reactions in antigen–antibody tests [48,49]. In addition, in a study from Benin, it was shown that COVID-19 Ab EIA reactive pre-pandemic malaria samples did not contain SARS-CoV-2-specific neutralizing antibodies [43]. Non-specific signals in LFA such as COVID-19 Ab RDTs can be reduced by engineering the components, e.g., adding blocking proteins to the buffer or adapting the capillary flow rate by changes in the nitrocellulose strip [13]. Of note, for both dengue and *P. falciparum* malaria, in silico evidence of similarity with SARS-CoV-2 epitopes has also been put forward to explain the cross-reactivity [50].

IgM test lines accounted for more than two-thirds (67.9%) of cross-reacting test lines. False-positive IgM test lines in COVID-19 Ab RDTs have been reported before [18,42,51], and given the comparable kinetics of IgM and IgG, selective testing for IgM has been questioned [52]. In that regard, a seroprevalence study in Spain took into account only the IgG results of the COVID-19 Ab RDT used, while disregarding the IgM results [53]. Doing so for the present panel would reduce the overall cross-reactivity by more than two-thirds, i.e., from 18.5% to 5.9%, and for some products more than 10-fold to below 4%. For schistosomiasis and dengue, disregarding the IgM line would eliminate the cross-reactivity in 9/13 and 12/13 RDT products, respectively (Supplementary Table S4).

A total of 30% of IgM and 40% of IgG cross-reactive test lines in the present study had at least weak line intensity, i.e., they were clearly visible. Nearly two-thirds of all cross-reactive test lines had low intensity, and one might consider to disregard them as negative in order to increase specificity. However, five RDT products explicitly prescribed to consider any shade of color as a positive reading. In addition, low intensity has been reported frequently among true-positive test lines in COVID-19 RDTs assessed in independent evaluation studies or deployed in seroprevalence studies in Europe [14,54,55,56,57,58,59]; in the latter studies, proportions of low intensity were comparable to the present cross-reactive line intensities [58,59]. In the case of seroprevalence studies, this may partly be explained by lower antibody levels in non-symptomatic individuals [60]. Consequently, considering low line intensities as negative would probably have a considerable cost of decreased sensitivity [61].

The present retrospective study had its limitations. It only assessed RDTs for cross-reactions. There were no control groups (e.g., healthy individuals or confirmed COVID-19 patients) for comparison, and in view of the ethical clearance it was not possible to exclude other potential causes of cross-reactivity or interfering substances such as the presence of rheumatoid factor, anti-nuclear antibodies, haemoglobin or concurrent malaria antibodies in schistosomiasis or dengue samples. Further, only a fraction of COVID-19 Ab RDTs marketed and one lot per product were assessed, whereas lot-to-lot variations may occur [8]. In view of limited accessible sample volumes, extra COVID-19 diagnostic testing (e.g., with other EIA platforms) was not possible. Test results were only read at the end of the readout time, which may have slightly decreased specificity [62]. Further, tests were carried out by laboratory technicians experienced in RDT evaluations, which may have increased the observations of very faint test lines. Finally, the retrospective nature of the present study did not allow the assessment of capillary blood, whereas the frozen blood used was not recommended as a specimen by seven RDT products.

Among the strengths, there was the large panel of geographically representative samples covering a selection of known cross-reacting diseases in a majority of non-hospitalized patients. Different malaria subgroups, *Plasmodium* species and parasite density groups were included. The selection of RDT products covered the different antigens used and had various regulatory approvals. Some of them were produced by reputable manufacturers and had been successfully validated in peer-reviewed studies [6,21,25,34,63,64,65,66] and approved by regulatory authorities (FDA EUA) and independent actors such as FIND [2]. In addition, the laboratory workflow was highly standardized and monitored. As an example, a micropipette was used instead of the product’s sample transfer device in order to minimize inaccurate results induced by variations in sample transfer. Further, the trained laboratory technicians had expertise in distinguishing very faint test lines from ghost lines (i.e., shade of lines caused by the application of the test line antibodies on the nitrocellulose strip).

For seroprevalence studies, test specificity is key [58] and this concerns sub-Saharan Africa in particular, where the prevalence of COVID-19 is low compared to other continents [67] and *P. falciparum* malaria-endemicity is high [68]. In such a context, most of the presently assessed RDT products would result in high numbers of false-positive results, probably outnumbering the true positive ones. Of note, the geographically more dispersed *P. vivax* was also associated with cross-reactivity, whereas WHO guidance documents mention to test cross-reactivity only for *P. falciparum* and *P. ovale* [31,32]. A similar impact can be expected in areas endemic for schistosomiasis and dengue. Adding to the challenges of seroprevalence studies with COVID-19 Ab RDTs is the use of capillary blood (the preferred specimen for seroprevalence studies in LMICs [10]), which needs further study as to its accuracy, particularly when used in field settings [6,8,13]. As to the applications of triage and COVID-19 disease diagnosis in LMICs, the similarity of its early clinical presentations with those of malaria and dengue and the potential co-occurrence of these diseases present additional problems [25,32,40,44,69].

Although not a study objective (and not systematically assessed), we observed shortcomings in products’ instructions for use (not mentioning the detecting antigen for 11/13 products), manufacturing (bacterial contamination of the buffer vial, errors of cassette assembly and labeling) and performance (weak control and test lines). These shortcomings can seriously affect the usability of the products in the field; they have been noted before and may be caused by rapid development in view of high customer demand and insufficient regulatory oversight [4,8,70].

Recommendations to mitigate risks of cross-reactivity in COVID-19 Ab RDTs have been made before: select a product with proven specificity for the co-endemic diseases, adjust epidemiological findings according to products’ accuracy, consider only the IgG test line result, combine results of two independent products and perform confirmatory EIA testing [41,53,71,72]. Further, as discussed above, product engineering can optimize a product’s specificity [13]. In view of experiences with other RDTs, human African trypanosomiasis, leishmaniasis and Chagas disease are other co-endemic diseases that may cause cross-reactivity with COVID-19 Ab RDTs [29,38]; based on a previous study, acute Zika infection should be considered as well [16]. HIV infection so far has not been associated with cross-reactivity in COVID-19 Ab RDTs [17,19,22,26]. COVID-19 Ab EIA and CLIA have higher sensitivity and specificity than RDTs [4,62,73], but the cross-reactivity of the DiaPro EIA in the present study reminds us that EIA can also be affected. Based on laboratory reference testing, the best performing COVID-19 Ab RDTs should be assessed prospectively for accuracy and usability in the real-life setting of the target population and its co-endemic diseases [41,42,43,74].

## Figures and Tables

**Table 1:** Overview of different COVID-19 antibody detecting products and EIA according to manufacturer, targeted antigens (S = spike, N = nucleocapsid, RBD = receptor binding domain) and different test lines (IgG and/or IgM). Underlined words indicate the abbreviation used in the text. EIA refers to enzyme immunoassay. Wantai RDT product has a single test line, no information was given about the class of antibodies detected (ND = no data). Claimed specimen: S = serum, P = plasma, WB = whole blood.

RDT Products	Manufacturer	Detecting Antigen	IgG/IgM	Claimed Specimen
Wantai SARS-CoV-2 Ab Rapid Test	Wantai Bio-Pharm	S	ND	S/P/WB *
COVID-19 IgG/IgM Rapid Test Cassette	Healgen	S	IgG/IgM	S/P/WB *
TODA CORNODIAG +	Todapharma **	N	IgG/IgM	S/P/WB
Rapid 2019-nCOV IgG/IgM Combo Test Card	Boson Biotech	N	IgG/IgM	S/P/WB
SARS-CoV-2 IgM/IgG Antibody Test Kit	Biohit	N	IgG/IgM	S/P/WB
Panbio COVID-19 IgG/IgM Rapid test device	Abbott **	N	IgG/IgM	S/P/WB *
QuickZen COVID-19 IgM/IgG	ZenTech **	RBD	IgG/IgM	S/P/WB *
StrongStep SARS-CoV-2 IgM/IgG Antibody Rapid Test	Liming Bio	N + S	IgG/IgM	S/P/WB *
2019-nCOV IgG/IgM Rapid Test	Dynamiker	N + S	IgG/IgM	S/P/WB
qSARS-COV-2 IgG/IgM Rapid Test	Cellex **	N + S	IgG/IgM	S/P/WB
COVID-19 IgG/IgM Rapid Test Cassette	SureScreen Diagnostics **	N + RBD	IgG/IgM	S/P/WB *
COVID-19 IgG/IgM Detection Kit (Colloidal Gold)	Singuway	N + RBD	IgG/IgM	S/P/WB
COVID-19 IgM/IgG Ab Test Cassette	Multi-G	N + RBD + S	IgG/IgM	S/P/WB *
**Comparator EIA**				
2019 nCOV IgG SPIKE EIA	Launch Diagnostics Limited/ DiaPro IgG EIA	S	IgG	S/P *
2019 nCOV IgM EIA	Launch Diagnostics Limited/ DiaPro IgM EIA	N + S	IgM	S/P *

Products with “*” mentioned in the instructions for use not to use frozen whole blood to perform RDT testing. Products with “**” refer to those for which instructions for use explicitly mentioned to read “any shade of color” as a positive test line.

**Table 2:** Definitions, total numbers of sample specimens (EDTA whole blood/serum), origin, patient type, age and gender per disease. Reference testing and definitions for malaria, dengue and schistosomiasis samples were based on previous studies [27,35,36,37]. Abbreviations: HRP-2 Ag = histidine-rich protein 2 antigen, IFA = immunofluorescence assay, IQR = inter-quartile range, EIA = enzyme immunoassay, IHA = indirect hemagglutination assay, VFR = visiting friends and relatives.

Infection and Subgroup		Definition and Description	Samples (*n* = 220)									Patients (*n* = 196)		
			Specimen		Geographic Origin				Patient Type			Age	% Children (<15 years)	% Females
			EDTA Whole Blood	Serum	African States	Asia-Pacific States	Latin America and Caribbean States	No data	Expatriates and Travelers	VFR and Migrants	Not Specified	Median (IQR)		
Malaria (*n* = 170)	Current malaria	Positive microscopy (thick and thin blood film) AND Confirmation by in-house real-time PCR See Table 4 for details of species identification and parasite density	110	16	101	8	-	17	21	35	70	36.5 (26–47.5)	9	37
	Recent malaria	Any of the following: 1. Clinical information AND detectable antibodies (*n* = 12) 2. HRP-2 Ag positive, PCR positive; microscopy negative (*n* = 9) 3. Repeat microscopy after positive thickblood film + presence of gametocytes (*n* = 1)	8	13	21	-	-	-	10	9	2	42.5 (34–51)	0	35
	Malaria antibody	Positive IFA titer (> 1/80) for at least 1 out of 4 *Plasmodium* species	1	22	21	-	-	2	2	19	2	42 (23–60.5)	9	18
Schistosomiasis (*n* = 25)		EIA (*Schistosoma mansoni* ELISA kit (Bordier Affinity Products)) and IHA positive result (ELI.H.A Schistosoma kit (ELITechGroup Microbiology)) AND ≤ 30 year (*n* = 25 ) All patients had negative (*n* = 8) or unknown (*n* = 17) result for dengue and malaria and were in chronic stage of disease A total of 11 patients had a positive microscopy for Schistosoma eggs in feces	2	23	24	1	-	-	15	5	5	13 (7.5–21.5)	61	57
Dengue (*n* = 25)		< 4 weeks after symptom onset AND IgG (Dengue Virus IgG Dx Select (Focus Diagnostics)) and IgM EIA (Dengue Virus IgM Capture Dx Select (Focus Diagnostics)) All patients had negative (*n* = 3) or unknown (*n* = 22) result for schistosomiasis and malaria	5	20	6	10	9	-	12	9	4	39 (24–50)	0	30
Total			126	94	173	19	9	19	60	77	83	35 (24–47)	13	37

**Table 3:** Cross-reactions according to disease groups and specimen types for the different RDT products and comparator enzyme immunoassay (EIA). Due to numbers of invalids and availability of samples, numbers of samples tested per product and disease combination may vary slightly. All data are %, unless otherwise stated. Invalid results are subtracted, and 95% C.I. (= Confidence Interval) is calculated for all diseases combined per RDT product. Abbreviations: NA = not applicable.

Product	Detecting Antigen	Diseases								Specimen	
		Malaria Total	Current Malaria	Recent Malaria	Malaria Antibody	Dengue	Schistosomiasis	All Diseases Combined	95% C.I for all Diseases Combined	Serum	Whole Blood
RDTs: numbers of samples tested (*n*)		170	126	21	23	25	25	220	220	94	126
Toda	N	3.5	1.6	4.8	13.0	-	-	2.7	1.0–5.8	5.3	0.8
Cellex	N + S	4.2	2.4	20.0	0.0	5.0	-	3.8	1.4–8.1	6.8	1.2
Multi-G	N + RBD + S	7.6	5.6	19.0	8.7	-	-	5.9	3.2–9.9	1.1	9.5
SureScreen	N + RBD	5.9	6.3	-	8.7	4.0	16.0	6.8	3.9–11.0	9.6	4.8
StrongStep	N + S	9.6	10.4	10.0	4.5	4.0	4.0	8.3	5.0–12.8	8.5	8.1
QuickZen	RBD	11.2	9.5	14.3	13.0	8.0	20.0	11.4	7.5–16.3	21.3	4.0
Biohit	N	16.5	14.3	14.3	30.4	4.0	12.0	14.5	10.2–19.9	21.3	9.5
Singuway	N + RBD	15.3	11.1	28.6	26.1	-	60.0	18.6	13.7–24.4	33.0	8.1
Panbio	N	25.3	25.4	19.0	30.4	4.0	4.0	20.5	15.3–26.4	20.2	20.6
Dynamiker	N + S	30.6	24.6	47.6	47.8	16.0	20.0	27.7	21.9–34.1	40.4	18.3
Healgen	S	27.8	24.8	42.9	26.1	8.0	76.0	30.6	24.6–37.2	42.6	21.6
Wantai	S	45.3	54.8	28.6	8.7	12.0	4.0	36.8	30.4–43.6	3.2	61.9
Boson	N	56.2	53.6	66.7	60.9	32.0	16.0	48.9	42.1–55.7	54.3	44.5
All RDTs combined		20.3	19.2	24.4	21.6	7.5	18.1	18.5	17.1–20.0	20.8	16.8
EIA: numbers of samples tested (*n*)		68	30	16	22	20	23	111	111	94	17
DiaPro IgG EIA	S	2.9	3.3	6.3	-	-	-	1.8	0.2–6.4	-	11.8
DiaPro IgM EIA	N + S	19.1	20.0	25.0	13.6	15.0	8.7	16.2	9.9–24.4	17.0	11.8

**Table 4:** Details of cross-reactions (CR) for the RDT products and the current malaria disease group. Numbers and proportion of cross-reactions according to the different *Plasmodium* spp. and, for *Plasmodium falciparum*, parasite density groups are expressed as numbers of asexual parasites/µL. For each RDT product, the detecting antigens are listed: S = spike protein, N = nucleocapsid protein, RBD = receptor binding protein. Abbreviations: CR = cross-reactions and EIA = enzyme immunoassay.

Malaria Species	Total Numbers Tested	Total Number of CR for Any Product	No CR in Any Product	Wantai (S)	Healgen (S)	Toda (N)	Boson (N)	Biohit (N)	Panbio (N)	QuickZen (RBD)	StrongStep (N + S)	Dynamiker (N + S)	Cellex (N + S)	SureScreen (N + RBD)	Singuway (N + RBD)	Multi-G (N + RBD + S)	DiaPro IgG EIA (S)	DiaPro IgM EIA (N + S)
*Plasmodium falciparum*	95	86	9	51	26	-	50	12	23	12	10	22	1	7	12	4	1	4
Pf 0–500	28	27	1	13	11	-	21	7	9	5	3	10	1	4	7	2	1	2
Pf 501–5000	23	22	1	14	6	-	10	2	4	1	2	4	-	1	1	-	-	1
Pf > 5001	44	37	7	24	9	-	19	3	10	6	5	8	-	2	4	2	-	1
*Plasmodium vivax*	11	11	-	8	2	-	5	1	1	-	1	2	-	-	-	1	-	-
*Plasmodium ovale*	12	9	3	7	3	-	7	-	1	-	-	1	-	-	1	1	-	1
*Plasmodium malariae*	8	7	1	3	1	2	5	5	7	-	1	6	1	1	1	1	-	1
Total	126	113	13	69	32	2	67	18	32	12	12	31	2	8	14	7	2	6

## Data Availability

The database for this manuscript will be made open access. Access requests for ITM research data can be made to ITM’s central point for research data access by means of submitting the completed Data Access Request Form. These requests will be reviewed for approval by ITM’s Data Access Committee (ITMresearchdataaccess@itg.be).

## Supplementary Materials

The following are available online at https://www.mdpi.com/article/10.3390/diagnostics11071163/s1, Table S1: Extra information of different COVID-19 antibody detecting products and EIA according to manufacturer, instructions for use and product regulation, Table S2: Classification of country of travel or residence per disease, traveler type and gender, Table S3: Pilot testing of the control samples and the invalid test rates per RDT product, Table S4: Cross-reactions according to the test lines affected and test line intensities for the different RDT products.
