# Supplementary material for: COVID-19 Antibody Detecting Rapid Diagnostic Tests Show High Cross-Reactivity When Challenged with Pre-Pandemic Malaria, Schistosomiasis and Dengue Samples
Source: Diagnostics (Basel). 2021 Jun 25;11(7):1163. doi: 10.3390/diagnostics11071163 (PMC8305106; doi:10.3390/diagnostics11071163)
Supplement: Supplementary file 1 [file diagnostics-11-01163-s001.zip › diagnostics-1264710-supplementary final.pdf]

Supplementary Material

COVID-19 Antibody Detecting Rapid Diagnostic Tests Show High Cross-Reactivity When Challenged with Pre-Pandemic Malaria, Schistosomiasis and Dengue Samples

Fien Vanroye [1,*], Dorien Van den Bossche [1], Isabel Brosius [1], Bieke Tack [1,2], Marjan Van Esbroeck [1] and Jan Jacobs [1,2]

[1] Department of Clinical Sciences, Institute of Tropical Medicine, 2000 Antwerp, Belgium; dvandenbossche@itg.be (D.V.d.B.); ibrosius@itg.be (I.B.); btack@itg.be (B.T.); mvesbroeck@itg.be (M.V.E.); jjacobs@itg.be (J.J.)
[2] Department of Microbiology, Immunology and Transplantation, KU Leuven, 3000 Leuven, Belgium
* Correspondence: fvanroye@itg.be

Supplementary Table S1. Extra information of different COVID-19 antibody detecting products and EIA according to manufacturer, Instructions for Use and product regulation. Data are those obtained at selection and procurement (August-September 2020). Unless otherwise stated, data are those from the Instructions for Use or the labeling of the product. Wantai RDT product has a single test line, no information was given about the nature of the antibody detected (ND = no data). Claimed specimen: S = serum, P = plasma, WB = whole blood. Sensitivity and specificity results according to FIND independent SARS-CoV-2 Ab evaluations are found on https://www.finddx.org/sarscov2-eval-antibody/ (NA = not applicable). Products with "*" had no detecting antigen mentioned in the instructions for use, information was retrieved from the manufacturer or the FDA EUA or WHO EUL, unless otherwise stated (column antigen used for detection). Abbreviations: EIA = enzyme immunoassay, CE = Conformité Européenne (CE Mark), FDA EUA = U.S. Food and Drug Administration Emergency Use Authorization, WHO EUL = World Health Organization Emergency Use Listing, FAGG = Federal Agency for Medicines and Health Products, FIND = Foundation for Innovative New Diagnostics, IFU = Instructions for Use. Products with "**" did not mention the use of fingerstick blood as a possible specimen. Cellex recommend not to use fingerstick specimens, as it is not validated. Information about regulatory status was obtained during the procurement of the RDT products in August 2020. Products with "ᵻ" mentioned in the instructions for use not to use frozen whole blood to perform RDT testing.

**Product**	**Manufacturer**	**City (Country)**	**Antigen used for detection**	**IgG/IgM**	**Claimed specimen**	**Claimed sensitivity**	**Claimed specificity**	**Product code**	**Lot number**	**Shelf life upon reception (months)**	**IFU version**	**Date IFU**	**Price/test (in euros, taxes not included)**	**Regulatory authorization (CE, FDA, FAGG)**	**Listed on WHO EUL**	**FIND**	**Results FIND sensitivity**	**Results FIND specificity**
**Wantai SARS-CoV-2 Ab Rapid Test**	Wantai Bio-Pharm	Beijing (China)	S *	ND	S/P/WB ᵻ	99,7%	98,9%	WJ-2750	JNB20200304	6	20/04	26/04/2020	5,90	CE, FDA	Yes	Yes	Pending Finalization	
**COVID-19 IgG/IgM Rapid Test Cassette**	Healgen	Houston (USA)	S *	IgG/IgM	S/P/WB ᵻ	IgG:97,2% IgM: 87,9%	IgG:100% IgM: 100%	GCCCOV-402a	2003309	17	B21854-02	4/03/2020	11,25	CE	No	No	NA	NA
**TODA CORNODIAG +**	Todapharma	Strasbourg (France)	N *	IgG/IgM	S/P/WB	IgG:100% IgM:100%	IgG:100% IgM:100%	2275+/25	2005081A	19	Version 3	03/08/2020	9,45	CE	No	No	NA	NA
**Rapid 2019-nCOV IgG/IgM Combo Test Card**	Boson Biotech	Fujian (China)	N *	IgG/IgM	S/P/WB **	87,8%	99,0%	1N38C2	20031926	11	81985	20/03/2020	8,75	CE	No	Yes	NA	NA

**SARS-CoV-2 IgM/IgG Antibody Test Kit**	Biohit	Hefei (China)	N *	IgG/IgM	S/P/WB	IgG:97,5% IgM: 97,5%	IgG:100% IgM: 99,5%	NA	SA200401	6	Version02	22/02/2020	2,55	no CE, FDA	Yes	Yes	NA	NA
**PanBio COVID-19 IgG/IgM Rapid test device**	Abbott	Jena (Germany)	N *	IgG/IgM	S/P/WB Ɨ	97,8%	92,8%	ICO-T402	COV0052 023	7	NA	6/06/2020	6,00	CE, FAGG	No	No	Withdrawn	
**QuickZen COVID-19 IgM/IgG**	ZenTech	Angleur (Belgium)	RBD	IgG/IgM	S/P/WB Ɨ	IgG:100% IgM:97%	IgG:97% IgM:99,1%	SLW-25	SLW25-007A	18	S-LW-MZ-002	1/04/2020	9,80	no CE, FAGG	No	Yes	55.1%	99.0%
**StrongStep SARS-CoV-2 IgM/IgG Antibody Rapid Test**	Liming Bio	Jiangsu (China)	N + S *	IgG/IgM	S/P/WB ** Ɨ	IgG:93,3% IgM: 98,7%	IgG:98,7% IgM: 100%	502090	2006003	19	V4.0	30/05/2020	2,55	no CE, FAGG	No	Yes	NA	NA
**2019-nCOV IgG/IgM Rapid Test**	Dynamiker	Tianjin (China)	N + S	IgG/IgM	S/P/WB	93,2%	95,3%	DNK-1419-1	200501	12	NA	NA	11,00	CE, FAGG	No	Yes	69.7%	95.3%
**qSARS-COV-2 IgG/IgM Rapid Test**	Cellex	North Carolina (USA)	N + S *	IgG/IgM	S/P/WB **	93,8%	96%	5515C025	20210	9	NA	NA	17,00	CE	No	Yes	Withdrawn	
**COVID-19 IgG/IgM Rapid Test Cassette**	SureScreen Diagnostics	Derby (UK)	N + RBD *	IgG/IgM	S/P/WB Ɨ	IgG:100% IgM: 91,8%	IgG:99,5% IgM: 99,2%	COVID19C	COV2004 0037	17	RP327102	5/03/2020	7,36	CE, FAGG	No	No	NA	NA
**COVID-19 IgG/IgM Detection Kit (Colloidal Gold)**	Singuway	Shenzhen (China)	N + RBD *	IgG/IgM	S/P/WB **	≥95-100%	100%	NA	200404A	no expir y date	402-000032-00V1.2en	28/03/2020	9,50	no CE	No	No	NA	NA
**COVID-19 IgM/IgG Ab Test Cassette**	Multi-G	Antwerpen (Belgium)	N + RBD + S *	IgG/IgM	S/P/WB ** Ɨ	IgG:93% IgM: 82%	IgG:96% IgM: 97,5%	MGS	COV1252 006A	8	Rev01 20200623	23/06/2020	5,00	CE	No	Yes	NA	NA

**2019 nCOV IgG SPIKE EIA**	Launch Diagnostics Limited/DiaPro IgG EIA	Milano (Italy)	S	IgG	S/P ‡	100%	>98%	COV19Gspike.CE/S	0720	12	rev. 0c	1/06/2020	350,00	CE	No	No	NA	NA
**2019 nCOV IgM EIA**	Launch Diagnostics Limited/DiaPro IgM EIA	Milano (Italy)	N + S	IgM	S/P ‡	98%	98%	COV19M.CE.192/S	0420/2AA	9	rev. 2c	1/06/2020	840,00	CE	No	No	NA	NA

Supplementary Table S2. Classification of Country of Travel or Residence per disease, traveler type and gender.
Countries are classified according to the United Nations Statistics Division for Geographic Origins (unstats.un.org/unsd/methodology/m49/).
Abbreviations: VFR = visiting friends & relatives.

Country of Travel or Residence				Disease (samples)				Traveler Type (patients)			Gender (patients)	
				Malaria (*n* = 153)	Dengue (*n* = 20)	Schistosomiasis (*n* = 23)	Total disease (*n* = 196)	Expatriates & Travelers (*n* = 51)	VFR & migrants (*n* = 66)	No data (*n* = 79)	Male (*n* = 124)	Female (*n* = 72)
Africa	Northern Africa		Morocco	1	-	-	1	-	-	1	1	-
			Sudan	1	-	1	2	1	1	-	2	-
			Total	2	-	1	3	1	1	1	3	-
	Sub-Saharan Africa	Eastern Africa	Burundi	1	-	2	3	2	-	1	-	3
			Eritrea	1	-	1	2		2	-	2	-
			Ethiopia	3	-	1	4	1	3	-	3	1
			Kenya	1		-	1	-	-	1	-	1
			Madagascar	1	-	-	1	1	-	-	1	-
			Malawi	1	-	-	1	1	-	-	1	-
			Mozambique	1	-	-	1	-	-	1	1	-
			Rwanda	2	-	6	8	6	2	-	4	4
			Somalia	-	1	-	1	1	-	-	1	-
			Uganda	3	-	-	3	1	2	-	1	2
			United Republic of Tanzania	1	-	-	1	1	-	-	1	-
			Total	15	1	10	26	14	9	3	15	11

Africa	Sub-Saharan Africa	Middle Africa	Angola	1	1	1	3	1	2	-	2	1
			Cameroon	16	-	-	16	-	5	11	10	6
			Central African Republic	1	-	-	1	1	-	-	1	-
			Chad	2		-	2	2	-	-	1	1
			Congo	-	1	-	1	-	1	-	1	-
			Democratic Republic of the Congo	28	-	-	28	8	12	8	17	11
			Gabon	1	-	1	2	-	-	2	-	2
			Total	49	2	2	53	12	20	21	32	21
		Western Africa	Benin	2	-	-	2	1	-	1	2	-
			Burkina Faso	6	-	-	6	1	2	3	4	2
			Côte d'Ivoire	5	1	-	6	2	2	2	4	2
			Ghana	16	-	4	20	6	5	9	11	9
			Guinea	10	-	2	12	-	8	4	9	3
			Liberia	3	-	1	4	-	4	-	2	2
			Mali	2		1	3	-	1	2	2	1
			Nigeria	7	-	-	7	-	7	-	5	2
			Senegal	5	1	1	7	1	1	5	5	2
			Sierra Leone	2	-	-	2	-	-	2	1	1
			Togo	3	-	-	3	-	2	1	2	1
			Total	61	2	9	72	11	32	29	47	25

Americas	Latin America and Caribbean	Caribbean	Cuba	-	1	-	1	1	-	-	-	1
			Haiti	-	2	-	2	1	1	-	1	1
		Central America	Mexico	-	1	-	1	1	-	-	-	1
			Nicaragua	-	1	-	1	1	-	-	-	1
		South America	Ecuador	-	1	-	1	-	-	1	-	1
			Suriname	-	1		1	1	-	-	1	-
		Total		0	7	0	7	5	1	1	2	5
Asia	South-eastern Asia		Cambodia	1	1	-	2	-	-	2	1	1
			Indonesia	-	1	-	1	1	-	-	1	-
			Lao People's Democratic Republic	-	-	1	1	1	-	-	-	1
			Philippines	-	1	-	1	1	-	-	1	-
			Thailand	-	2	-	2	1	-	1	2	-
	Southern Asia		India	6	2	-	8	3	-	5	6	2
			Pakistan	1	-	-	1	-	-	1	1	-
			Sri Lanka	-	1	-	1	-	1	-	1	-
	Total			8	8	1	17	7	1	9	13	4
No data				17	-	-	17	1	2	14	11	6

Supplementary Table S3. Pilot testing of the control samples and the invalid test rates per RDT product.
For Cellex RDT product, only 158 samples were tested; for the DiaPro IgG & IgM EIA product, 94 serum and 17 plasma samples were tested.
Pilot testing: commercial controls were Multichem ID-COVID19G/M control and Multichem ID-COVID19 Neg (ZenTech, Angleur, Belgium). Result (pos/neg) and ratio are given for the patient control and commercial controls tested with DiaPro IgG and IgM EIA. Positive serum patient control was confirmed by in-house SARS-CoV-2 virus neutralization, negative serum patient control was a healthy person tested negative by WANTAI SARS-CoV-2 Ab ELISA (Wantai, Beijing, China). Abbreviations: EIA = enzyme immunoassay Results for IgG and IgM lines are presented as negative (N) and by line intensity: very faint (VF), faint (F), weak (W), medium (M) and strong (S). Invalid testing: types of invalid test results include: red background obscuring the test line (ORB), incomplete migration (MI), no control line visible (NCL), failed migration (FM), patchy broken test line (PL) and strip misplaced in cassette (SM) according to WHO round testing 2018 (https://www.who.int/malaria/publications/atoz/9789241514965/en/).

Product	Number of samples tested	Number of cross-reactions	Pilot testing control samples								Numbers (%) of invalid test results	Type of invalid test result (numbers)					
			Negative patient control		Positive Patient control		Multichem negative control		Multichem positive control			ORB	MI	NCL	FM	PL	SM
**Wantai**	220	85	N		M		N		W		1 (0.5%)	-	-	-	-	1	-
**Healgen**	219	67	N	N	F	S	N	N	S	W	2 (0.9%)	2	-	-	-	-	-
**Toda**	220	10	N	N	F	VF	N	N	F	VF	0	-	-	-	-	-	-
**Boson**	219	116	N	N	M	F	VF	N	M	VF	3 (1.4%)	-	-	-	-	3	-
**Biohit**	220	34	N	N	W	W	N	N	F	VF	0	-	-	-	-	-	-
**Panbio**	220	39	N	N	M	N	N	N	W	N	0	-	-	-	-	-	-
**QuickZen**	220	24	N	N	VF	M	N	N	VF	VF	42 (19.1%)	39	1	-	-	2	-
**StrongStep**	217	13	N	N	W	W	N	N	N	N	0	-	-	-	-	-	-
**Dynamiker**	220	73	N	VF	W	W	VF	VF	W	W	8 (3.6%)	5	1	-	-	-	2
**Cellex**	158	7	N	N	M	W	N	N	W	N	7 (4.4%)	-	-	7	-	-	-
**SureScreen**	220	12	N	N	W	M	N	N	S	VF	5 (2.3%)	5	-	-	-	-	-
**Singuway**	220	44	N	N	N	M	N	N	F	F	0	-	-	-	-	-	-
**Multi-G**	220	12	N	N	W	VF	N	N	W	N	4 (1.8%)	3	-	-	-	25	-
**DiaPro IgG EIA**	111	2	neg (0.7)	-	pos (3.2)	-	neg (0.74)	-	pos (5.99)	-	NA	-	-	-	-	-	-
**DiaPro IgM EIA**	111	18	-	neg (0.5)	-	pos (1.1)	-	pos (3.16)	-	pos (4.74)	NA	-	-	-	-	-	-

**Total samples**	3015	556	-	-	-	-	-	-	-	-	72 (2.4%)	54 (75.0%)	2 (2.8%)	7 (9.7%)	-	31 (43.1%)	2 (2.8%)
**Total serum samples**	1390	267	-	-	-	-	-	-	-	-	1 (0.07%)	-	1 (100%)	-	-	-	-
**Total whole blood samples**	1625	270	-	-	-	-	-	-	-	-	71 (4.4%)	54 (76.1%)	1 (1.4%)	7 (9.9%)	-	31 (43.7%)	2 (2.8%)

Supplementary Table S4. Cross-reactions according to the test lines affected and test line intensities for the different RDT products.
Data of test line reactivity are those obtained by consensus reading; data about line-intensity are those scored by observer 1. All data are %, unless otherwise stated. Invalid results are subtracted. Proportions of cross-reactions for Wantai were given as total Ig, proportions of intensities of cross-reactive lines were very faint (96.5%, faint (2.4%) and medium (1.2%) (mentioned with "*")). Abbreviations: NA = not applicable.

Product	Detecting antigen	Test lines affected					Line intensity (read by observer 1)											
							IgG						IgM					
		Nr of cross-reactions	**IgG only**	**IgM only**	**Total Ig**	**IgG+IgM**	*Nr of cross-reactive lines*	**very faint**	**Faint**	**Weak**	**Medium**	**Strong**	*Nr of cross-reactive lines*	**Very faint**	**Faint**	**Weak**	**Medium**	**Strong**
**Toda**	N	*10*	10.0	60.0	NA	30.0	*4*	25.0	25.0	50.0	-	-	*6*	66.7	16.7	16.7	-	-
**Cellex**	N + S	*7*	42.9	42.9	NA	14.3	*4*	25.0	50.0	25.0	-	-	*3*	66.7	33.3	-	-	-
**Multi-G**	N + RBD + S	*12*	16.7	83.3	NA	-	*2*	-	-	50.0	50.0	-	*10*	60.0	40.0	-	-	-
**SureScreen**	N + RBD	*12*	8.3	91.7	NA	-	*1*	-	100.0	-	-	-	*11*	72.7	9.1	18.2	-	-
**StrongStep**	N + S	*13*	7.7	92.3	NA	-	*1*	-	-	100.0	-	-	*12*	58.3	16.7	25.0	-	-
**QuickZen**	RBD	*24*	8.3	87.5	NA	4.2	*3*	100.0	-	-	-	-	*21*	47.6	33.3	19.0	-	-
**Biohit**	N	*34*	-	91.2	NA	8.8	*3*	33.3	-	66.7	-	-	*31*	48.4	19.4	32.3	-	-
**Singuway**	N + RBD	*44*	-	93.2	NA	6.8	*3*	33.3	66.7	-	-	-	*41*	41.5	31.7	26.8	-	-
**Panbio**	N	*39*	30.8	59.0	NA	10.3	*16*	25.0	25.0	31.3	18.8	-	*23*	39.1	39.1	21.7	-	-
**Dynamiker**	N + S	*73*	4.1	84.9	NA	11.0	*11*	63.6	9.1	27.3	-	-	*62*	56.5	16.1	24.2	3.2	-
**Healgen**	S	*76*	15.8	68.4	NA	15.8	*24*	37.5	20.8	25.0	8.3	8.3	*52*	46.2	25.0	19.2	7.7	1.9
**Wantai**	S	*85**	NA	NA	100.0	NA	*NA*	NA	NA	NA	NA	NA	*NA*	NA	NA	NA	NA	NA
**Boson**	N	*116*	6.0	84.5	NA	9.5	*18*	50.0	11.1	33.3	5.6	-	*98*	32.7	23.5	26.5	17.3	-
**All RDTs combined**		*545*	8.1	67.9	15.6	8.4	*90*	40.0	20.0	30.0	7.8	2.2	*370*	45.7	24.3	23.5	6.2	0.3
